# Exploring the long‐term psychosocial impact of paediatric haematopoietic stem cell transplantation for nonmalignant diseases

**DOI:** 10.1111/hex.13565

**Published:** 2022-07-29

**Authors:** Joëll E. Bense, Lieke ter Welle, Hilda Mekelenkamp, Marieke Schimmel, Marloes Louwerens, Arjan C. Lankester, Arwen H. Pieterse, Anne P. J. de Pagter

**Affiliations:** ^1^ Department of Pediatrics, Willem‐Alexander Children's Hospital, Leiden University Medical Center Division of Stem Cell Transplantation Leiden The Netherlands; ^2^ Department of Pediatrics, Willem‐Alexander Children's Hospital, Leiden University Medical Center Division of Psychosocial Care Leiden The Netherlands; ^3^ Department of Internal Medicine Leiden University Medical Center Leiden The Netherlands; ^4^ Department of Biomedical Data Sciences Leiden University Medical Center Leiden The Netherlands

**Keywords:** haematopoietic stem cell transplantation, late effects, nonmalignant diseases, paediatric, psychosocial impact

## Abstract

**Introduction:**

An understanding of the long‐term psychosocial impact of paediatric haematopoietic stem cell transplantation (HSCT) for nonmalignant diseases is needed to optimize pre‐HSCT counselling, supportive care and long‐term follow‐up programmes after HSCT for this group of patients and caregivers.

**Methods:**

This qualitative study included 14 patients who underwent transplantation for a nonmalignant disease during childhood. In‐depth interviews were held online to explore patients' perspectives on the long‐term psychosocial impact of HSCT on their lives. The results were analysed based on the Grounded Theory approach.

**Results:**

Patients' median age at the time of the interview was 19 years (range: 14–49), and the median years after HSCT was 12 years (range: 3–33). Four main themes were identified: (1) doing okay, (2) experiencing persistent involvement with healthcare services, (3) influence on relationships with loved ones and (4) impact on the patient's life course. Subthemes extracted were doing okay, feeling of being cured, health limitations, sense of vulnerability, ongoing connection to the hospital, acceptance, friendship, family relations, development of own identity, not taking life for granted, social development, impact on (school) career and thinking about the future.

**Conclusions:**

Patients reported active coping strategies and resilience after this high‐impact treatment. The data highlight the need for patient‐adjusted supportive care, indicating more need for supportive care in the long‐term outpatient clinic.

**Patient Contribution:**

This study included patients as participants. Caregivers were approached if patients were below a certain age. Additionally, preliminary results were presented during a patient conference day.

## 1 INTRODUCTION

Paediatric allogeneic haematopoietic stem cell transplantation (HSCT) is a curative treatment option for various malignant and nonmalignant diseases.^1^ The list of indications for paediatric HSCT for nonmalignant diseases is increasing and is commonly divided into three categories: haematologic (e.g., severe aplastic anaemia, sickle cell disease), metabolic (e.g., Hurler's disease) or immunological (e.g., severe combined immunodeficiency) diseases.^2^ Some of these diseases are life‐threatening; others are life shortening and decrease quality of life. Due to ongoing advances in HSCT procedures and concomitant improvements in survival, the long‐term physical and psychosocial outcomes of HSCT are becoming increasingly important.^3^, ^4^ While the psychosocial outcomes of HSCT for malignant indications have been characterized, data are scarce for patients with a nonmalignant disease.^5^, ^6^, ^7^, ^8^ The most frequently described long‐term psychosocial effects of HSCT for childhood cancer are anxiety, depression, posttraumatic stress reactions and behavioural and social problems. Both mental and physical late effects are likely to have a negative impact on patients' quality of life.^5^, ^7^, ^9^, ^10^, ^11^ Many children with nonmalignant diseases have already been coping with morbidity and chronic disease in the years before HSCT, often resulting in impaired quality of life.^12^, ^13^ HSCT is generally well known as a curative treatment for malignant diseases; this does not, however, apply to nonmalignant diseases, resulting in lack of peer support for these patients. There are no separate patients' associations for HSCT patients with nonmalignant diseases, as there are for patients with paediatric cancer.

A better understanding of the long‐term psychosocial impact of undergoing paediatric HSCT for nonmalignant diseases is needed to optimize pre‐HSCT counselling, supportive care and long‐term follow‐up programmes after HSCT for this group of patients. In this study, we explored patients' perspectives on the long‐term psychosocial impact of HSCT on their lives.

## 2 METHODS

### 2.1 Study design and participants

A qualitative descriptive design was used to determine patients' perspectives on the long‐term psychosocial impact of HSCT on their lives. In‐depth interviews were held by videoconference, due to COVID‐19 restrictions, from April to May 2021. Patients were interviewed one‐on‐one, and invited to be accompanied by their caregivers if they preferred. Companions were instructed to interfere as little as possible. The researcher conducted the interview using a topic guide consisting of open questions (Supporting Information: Table S1). All interviews were video‐recorded and transcribed verbatim. Field notes were taken about the researchers' reflections on the interview themes. Data collection continued until data saturation was reached, which was defined as no new findings emerging in the analysis of the three latest consecutive interviews. The research team consisted of four researchers with expertize in the HSCT field and qualitative research: L. t. W. (BSc, Master student, Medicine), J. B. (MSc, PhD candidate paediatrics), A. h. P. (PhD, cognitive psychologist) and A. d. P. (MD, PhD, paediatrician‐haematologist). All interviews were performed by an independent researcher (L. t. W.), who did not have any (treatment) relationship with the participants. This was made clear to the participants before the start of the interviews. The study protocol was approved by the medical ethics committee of Leiden University Medical Center (N20.181). Participants were approached by telephone (J. B.), had received complete study information and provided written informed consent. In the case of patients who were 15 years of age or younger, additional assent was provided by (both) caregivers.

All patients who underwent an HSCT during childhood for a nonmalignant disease in the Willem Alexander Children's Hospital in Leiden, The Netherlands, were eligible to participate in this study. Further eligibility criteria included HSCT 2 or more years ago, being ≥12 years of age at the time of the interview and having adequate knowledge of the Dutch or English language. Patients were selected using purposeful sampling based on age (age categories 12–18, 18–25, >25) and diagnosis (inborn errors of immunity, bone marrow failures, haemoglobinopathies).

### 2.2 Data analysis

Interviews were thematically analysed to find common patterns using the comprehensive 10‐step method of the Qualitative Analysis Guide of Leuven (Supporting Information: Table S2), which is based on the Grounded Theory associated with Charmaz.^14^, ^15^, ^16^, ^17^, ^18^, ^19^, ^20^, ^21^ The whole process consisted of constant comparison and continuous checking of the interview data. Each transcript was analysed by two independent researchers. The researcher who conducted the interviews was a permanent part of the data collection and analysis process. The other researchers on the team (J. B., A. h. P., A. d. P.) alternated in the role of second coder and analyser of the transcripts. Each step was first performed by the researchers individually, after which they came together to compare their findings and discuss discrepancies until a consensus was reached about key storylines, coding fragments, categorizing concepts and interpreting the data. Data collection and analysis took place parallel to each other. Final coding was entered into the qualitative data analysis software ATLAS.ti (version 8).^22^ In addition, the COREQ checklist for qualitative studies was used for explicit and comprehensive reporting (Supporting Information: Table S3).^23^

## 3 RESULTS

Eighteen participants were approached, and fourteen participants were willing to participate, with an equal gender distribution. Ages ranged from 14 to 49 years, and the interview took place at a median of 12 years (range: 3–33) after HSCT. Six participants were long term (2–10 years) after HSCT, and eight were very long term (>10 years) after HSCT. Indications for HSCT were inborn errors of immunity (*n* = 4), haemoglobinopathies (*n* = 4) or bone‐marrow failures (*n* = 6) (Table 1). The median interview duration was 35 min (range: 27–57). One participant preferred company from an adult caregiver. From the coding and categorizing of data, four main themes on psychosocial impact of paediatric HSCT emerged: (1) *Doing Okay*, (2) *Experiencing persistent involvement with healthcare services*, (3) *Influence on relationships with loved ones* and (4) *Impact on participant's life course*. Illustrative quotations are given per theme (Tables 2, 3, 4, 5).

**Table 1:** Patient characteristics (*N* = 14)

Characteristics	Median (range)
Gender	
Male	7
Female	7
Age at HSCT (in years)	10 (1–18)
Age at interview (in years)	19 (14–49)
Years since HSCT	12 (3–33)
Diagnosis^a^	
Inborn errors of immunity	4
Haemoglobinopathies	4
Bone marrow failures	6
Second HSCT	2

Abbreviation: HSCT, haematopoietic stem cell transplantation.

^a^ Conditioning regimens were busulphan‐based (*n* = 3), treosulphan‐based (*n* = 4), cyclophosphamide + TBI/TAI (*n* = 2), cyclophosphamide‐based (*n* = 3), fludarabine‐based (*n* = 1), no conditioning (*n* = 1). In the case of multiple HSCTs, the conditioning regimen of the first HSCT is reported.

### 3.1 Doing okay

#### 3.1.1 Doing okay

Almost all participants reported that they were ‘doing okay’ in their daily life. The participants were able to live the life they wanted and were feeling good. Many participants experienced late effects or needed medical treatment at the time of the interview. At the same time, the participants reported hardly any inconvenience in daily life from the disease and transplantation‐related late effects.

#### 3.1.2 Feeling of being cured

The participants were positive about their recovery after HSCT. However, some participants mentioned that the recovery took a long time, and that they had experienced some setbacks (e.g., slow immune reconstitution, sequelae of graft‐vs.‐host disease). The majority of the participants reported considering themselves as cured from the original disease, and had been able to leave the HSCT procedure behind them at some point.

### 3.2 Experiencing persistent involvement with healthcare services

#### 3.2.1 Health limitations

The participants reported limitations in daily life due to late effects/complications of the HSCT. Multiple late effects were reported, such as loss of fertility, alopecia, skin abnormalities or growth abnormalities. Fatigue was one of the most frequently experienced side effects, mainly during the first few years after transplantation. All participants experienced physical, social and emotional limitations due to the side effects. As a consequence, some participants reported limitations in daily activities and needed medical care. In addition, the participants indicated that they had needed to make adjustments in daily life, and had had to learn and rebuild life skills after the transplantation (e.g., rebuilding physical health, and only gradually returning to school).

#### 3.2.2 Sense of vulnerability

The participants reported feeling more susceptible to health issues compared to their peers. A few participants still did not feel completely healthy and reported being afraid of the possibility of experiencing new complications or disease recurrence. The participants reported frequent hospital visits for follow‐up and were aware of the possible late effects. Additionally, the participants were warned of possible health hazards, such as a COVID‐19 infection. All these factors exacerbated the participants' sense of vulnerability. For example, one participant stopped pursuing her healthcare study after her physician had warned her about her increased susceptibility to a COVID‐19 infection.

#### 3.2.3 Ongoing link with the hospital

Many participants needed medical help after the HSCT (e.g., consulting a psychologist or social worker, undergoing trauma therapy, participating in online psychological self‐help programmes, having operations, being admitted to the hospital, undergoing additional hospital checks, getting revaccinations and undergoing fertility treatments). Help was frequently sought at the initiative of the participant or was offered by the hospital. Some participants indicated that, in retrospect, medical help after discharge from the hospital had been insufficient or wished they had sought help sooner. Furthermore, the participants visited the hospital frequently for check‐ups, used medication on a daily basis or regularly had memories about the HSCT and the hospital. Some participants admitted that they experience the physical examinations and additional tests at the hospital as unpleasant. A few participants mentioned not being compliant with the therapy, as they did not notice that it made any difference to their overall condition. Lastly, nearly all of the participants mentioned occasionally looking back on the HSCT and hospital admission. These memories were generally positive and pleasant. A few participants reported experiencing anger or fear while thinking back to the HSCT.

#### 3.2.4 Acceptance

The participants indicated having a degree of acceptance on various aspects related to HSCT. The participants got used to the regular check‐ups and examinations at the hospital, and some stated that it helped them to feel more certain about their physical status. Additionally, the participants reported accepting the side effects. A few participants reported feeling unique because of the side effects. Lastly, the participants accepted that they had received a transplantation and felt relieved from the burden of the original disease. The participants accepted the HSCT as part of themselves and of their lives.

### 3.3 Influence on relationships with loved ones

#### 3.3.1 Friends

For most participants, the HSCT did not affect current friendships. The participants felt supported by their friends, and after returning to school, most participants rejoined friends without problems. When encountering new friends, the participants informed them about the HSCT and current relevant side effects. One participant reported losing friends due to the HSCT.

#### 3.3.2 Family relations

Some participants experienced changes in family relations as a result of the HSCT. Family relations had become closer and more equal (e.g., parent–child equality). The participants underlined receiving a lot of family support, both during and after the HSCT. However, a few participants mentioned that family relations had been damaged or that they had become more dependent on their family due to the HSCT. Some of their family members were very concerned and protective in the first few years after transplantation. The participants sometimes experienced this as annoying, but generally understood it well. Some participants had received stem cells from a family member. The experiences and thoughts about family donor tissue varied greatly, ranging from experiencing it as nice and personal to the feeling of being indebted to that family member.

### 3.4 Impact on participants' life course

#### 3.4.1 Development of own identity

Some participants struggled with existential questions, for example, who they are without the disease. Furthermore, the participants mentioned wanting to be independent and to make their own choices without being restricted by the HSCT. A number of participants underlined feeling different from their peers sometimes. In addition, some participants mentioned that it took a while to get used to the idea of having received stem cells from an unknown donor, and how this had raised questions about their ‘self’.

#### 3.4.2 Not taking life for granted

Having undergone transplantation during childhood means that the participants had faced issues of life and death at a young age, and some reported taking life less for granted. The participants reported being grateful for their lives and health. A few participants reported being proud of life achievements, in view of the health challenges that they had faced. Moreover, some participants described the HSCT as life‐saving and were generally grateful for having received the HSCT.

#### 3.4.3 Social–emotional development

A much‐discussed topic included the (dreaded) reactions of others to the visible physical consequences of the HSCT, such as alopecia, low voice, skin abnormalities or growth abnormalities. The participants mentioned regularly receiving comments or questions, mostly from strangers, leading to feelings of insecurity. A few participants admitted to avoiding public places because of these reactions, which made them feel restricted in establishing relationships. Some participants felt unsure about whether they would be perceived to be attractive by (potential) partners. Moreover, due to (anticipated risk of) infertility, some participants were afraid to disappoint their partner. The participants felt uncertain about acceptance by new friends. Experiencing stress before returning to school or feeling unable to keep up in class had led to experiencing a social gap with classmates. Furthermore, some participants reported having difficulties expressing emotions, as they lagged behind in learning how to regulate their emotions.

#### 3.4.4 Impact on (school) career

Many participants did not advance to the next grade in the year they underwent HSCT and were frequently absent from school due to side effects (such as fatigue) in the first few years after HSCT. A few participants had felt restricted in terms of career options due to the HSCT. The fear of complications or relapse or simply being confronted with memories of the HSCT had discouraged them from making more challenging school choices or pursuing particular career choices (such as not aiming for a higher school level, or prematurely terminating a study).

#### 3.4.5 Thinking about the future

Most participants stated that their HSCT experience would not play a role in the future. Some reported concerns, for example, finding a job, given physical limitations, finding a partner, the extent to which late effects would remain and whether or not they will be able to have children. Some participants hoped that limitations due to late effects would diminish with time, or that goals could be achieved (e.g., improvement in physical health, being able to have children or to obtain a driver's license).

**Table 2:** Illustrative quotations from Theme 1 ‘Doing okay’

Subtheme	Sex	Age	Quotation
Doing okay	*♀*	16	‘Yes, I'm doing really well. Besides the use of medication of course, I do not longer suffer from the transplantation. Or it's not that I notice in my daily life that, well, I've been transplanted. No, I'm just doing well’.
	*♂*	26	‘Yes, I'm doing well. I am feeling comfortable in my own skin, and I don't experience consequences of the transplantation’.
Feeling of being cured	*♂*	14	‘When I got out of the hospital and I was able to do things again, I basically left it all behind and started doing the things I like’.
	*♂*	18	‘It just feels like nothing has ever happened. Like I'm completely cured’.
	*♂*	18	‘Due to the transplantation I am able to do a lot more things. I feel normal now. Before the transplantation I really liked doing sports, but I always felt limited. Now, I don't feel that way anymore. When I am exercising, I can see myself growing and getting stronger. That feels great. So, that's how the transplantation has affected me’.

**Table 3:** Illustrative quotations from Theme 2 ‘Experiencing persistent involvement with healthcare services’

Subtheme	Sex	Age	Quotation
Health limitations	*♀*	20	‘I was very tired for a long time, and I was not able to do all the things my peers were able to do. And that's still there every now and then. I really have to think about my daily schedule; when I'm doing X, Y, Z today, then I should skip this tomorrow, because otherwise it would be too tiring’.
	*♂*	14	‘The left side of my body was paralyzed and is still functioning worse than my right side. However, it's not problematic as I use my right side the most. I write with my right hand. Some of my fingers can't move individually, which is somewhat annoying. But besides, it doesn't really bother me anymore’.
Sense of vulnerability	*♀*	49	[About checkups in the hospital]—‘So, on the one hand it's nice it's all being monitored. On the other hand, it also stirs things up a lot. The week in advance I'm really, well, not upset, but caught up by it. I know that my body is not as strong as someone else's body without a transplantation. So, every check‐up I face with the thought “oh god, what will be it this time”’.
	*♀*	20	‘I've seen how fragile life can be and that's still stuck in my head. Still, the possibility of that happening again scares me’.
Ongoing connection to the hospital	*♀*	20	‘But what came up recently, is I have difficulties undergoing examinations in the hospital. The transplantation is [x] years ago and mentally, I'm done with it’.
	*♀*	49	[About unfulfilled desire for having children]—‘So I went to see a social worker a few years ago for support. At that time all my friends got pregnant, which was really intense. Despite new insights, it still remains a thing’.
Acceptance	*♂*	18	[About skin manifestations]—‘It is what it is, and it belongs to me. And at some point, I embraced it’.
	*♀*	16	[About checkups in the hospital]—‘Actually, it's all normal now. I got used to it and no longer I think “oh I have to go to the hospital”. It's just a normal part of my life right now’.

**Table 4:** Illustrative quotations from Theme 3 ‘Influence on relationships with loved ones’

Subtheme	Sex	Age	Quotation
Friendship	♀	49	‘Friends always visited me at the hospital. So eventually when you're back, you're just part of the group again. And it feels like nothing's happened’.
	♀	20	‘Especially because I've lost friends due to my overload of emotional baggage. And I can understand that, but that's kind of… […] When I make new friends it's just like “hey, I'm blind and I've had a stem cell transplantation”. And that's something which influences me every day. So, that's quite anxious’.
Family relations	♀	16	‘They're the best parents I could wish for. When I need their help, they're always there for me. They always support me with everything’.
	♂	28	‘But my mother is always concerned. She has always said “take care of yourself” or “no, don't go” or “don't do it, just stay home”’.
	♀	16	‘My parents told me a lot about how things went and what it was like. That really makes me emotional. Because when my mother talks about that time, she starts to cry. It makes me realize it actually is very serious’.
	♀	17	‘But, compared to now, we grew apart. We're no longer the family of before and during the transplantation. That's a pity, because, for example my sisters grew up too. They wanted to continue with their lives and moved out. And now, it's just that I notice my family is not as close as it was in the past’.
	♀	29	‘It's beautiful that my brother was my donor. So, that's nice, it's something personal. It would be different if it had been an unknown donor. So it's nice that my brother could do this as he was also very young at that time’.

**Table 5:** Illustrative quotations from Theme 4 ‘Impact on patient's life course’

Subtheme	Sex	Age	Quotation
Development of own identity	♀	17	‘I'm really trying to find my identity. I am looking for who I am without the disease or who I am without the process of transplantation’.
	♂	26	‘And that's a thing I learned this last year. That I should go my own way and not always do the things my mother does. That woke me up mentally and made me see I had to develop myself’.
	♀	16	‘Of course you have to catch your breath, because it is a completely different person inside you. I received the immune system of someone else and have to get used to that’.
Not taking life for granted	♂	18	‘I believe that as a kid you don't think about the consequences or the dark side of certain things. As the result of exposure, at some point, you start to think differently about those things. And you don't know how to deal with that yourself, because you're actually too young for that’.
	♀	29	‘You really have to be aware of the fact that no one has promised you a new day. So, that's something you must be aware of in life. Because yes, you're alive, but it also could have ended differently. So, that's really something I remind myself of frequently’.
Social development	♀	16	‘People look at me strangely, as if I am a strange creature. People don't think it's normal that I'm short which gives me the feeling of not belonging here. That makes me feel sad and then I just don't dare going outside anymore. I then prefer to stay at home and not meet up with people’.
	♂	28	‘It's hard when you get to know someone who wants to become a mother. It was very difficult to tell her it might not work out’.
	♀	40	‘That was a really scary thing, to return to my old school. I didn't know my classmates anymore and it felt like a new school again’.
	♀	20	‘Socially, I couldn't keep up very well. For example, I once in class heard two children behind me talking about 'oh we were at a party this weekend and the police came' and then I thought “are you proud of that?”. That just felt like a very big gap, that they were concerned about such things while I was focusing on the next time I had to visit the hospital to get my blood checked and whether that would be okay or not. That created a big gap between me and my classmates’.
Impact on (school) career	♀	16	‘Last year, my grades were pretty good and there was an opportunity to level up at school. Then we finally decided not to, because if I get sick for 2‐3 weeks this winter, would I still be able to handle it? Or with corona for example, I could maybe become a vulnerable group when it would get worse. Therefore, with choices I think twice about “would it be wise?”’.
	♂	14	‘And also, when I was allowed to get back to school again, it was very difficult to notice that I actually couldn't keep up as I was just too tired’.
(Worrying about) the future	♂	28	‘So uh, I only hope I have kids then. […] We only had an investigation once and then the doctors said the chance of getting pregnant in a natural way is not very big. But that was only one test, and with testing once, you will not always see all results. Yes, I think we have to do two more tests’.
	♀	16	‘Because, if I want to get married, I have to find a man my height because, I can't be with a man who is much taller than me. So, I'll just have to see how things go’.
	♂	14	‘As soon as my physical endurance is good again, I will no longer suffer from it’.

## 4 DISCUSSION

This study provides a qualitative exploration of the long‐term psychosocial impact among survivors of paediatric HSCT for nonmalignant disease. The literature to date mainly focuses on patients with malignant conditions. Moreover, approaches using quantitative measures (e.g., questionnaires) are dominant in this field, which do address possible psychosocial topics, but are insufficient to provide broader insight on the psychosocial burden. This study reveals four main life areas in which patients experience the psychosocial impact of the HSCT. First, patients indicate that they are *doing okay* and feel cured from their original disease. Second, patients continue to *experience involvement with healthcare services* their entire life. This is mainly due to persisting side effects, which negatively affect patients in their daily functioning, contribute to a sense of vulnerability and make them seek out medical support. Patients experience having a continuous attachment to the hospital, while at the same time, they downplay and accept side effects, check‐ups in the hospital and having experienced the HSCT to a large extent. Third, HSCT *changes family relations and friendships* both in positive and negative ways. Lastly, HSCT *interferes with patients' course of life* in terms of their social development, progress in (school) career, development of identity and how they see the future.

Our study reveals some similarities to what has been observed in childhood cancer patients treated with HSCT. These patients also experienced social problems, social withdrawal, physical problems, changes in family relations, fear of disease recurrence, the desire to been seen as normal and impact on (school) career. Other similarities are the acceptance of serious side effects, ongoing use of medication, regular hospital check‐ups and the feeling of being cured after HSCT.^5^, ^8^, ^24^, ^25^ The latter has also been reported among sickle cell disease patients after HSCT.^12^ A study on cancer patients who sought psychotherapeutic help after HSCT found that patients felt different, lost contact with friends and showed family dependency,^26^ which is similar to our results. In contrast to what was reported in the former studies, the patients in our study did not experience symptoms of anxiety, depression or ask themselves ‘what if I didn't have received the HSCT’.^5^, ^7^, ^24^ These complaints may be less common in patients with nonmalignant disorders, or may not have been experienced or expressed by the patients who participated in the interviews.

Unique psychosocial themes that emerged in our study include experiencing a continuous attachment to the hospital, not taking life for granted, the search for one's own identity and thinking about the future. Strikingly, a number of patients expressed remarkable contradictions between and within themes. Specifically, patients mentioned that they were doing okay (Theme 1), while also expressing feelings of vulnerability (Theme 2). In addition, patients indicated having a good relationship with friends, but sometimes also perceived a social gap with peers (Theme 3). Patients mentioned impactful limitations, such as experiencing a persisting connection with healthcare services, having to deal with late effects on a daily basis and the HSCT influencing their life course. It has to be noted that most patients tended to tone down the limitations they experience. Clearly, these limitations play a significant role in the patients' lives, but accepting them or trying to accept them has become part of their identity. The extent to which patients accept side effects and check‐ups at the hospital is remarkable. It indicates that patients have developed active coping strategies and resilience.

This study has a number of strengths. First, purposeful sampling of participants allowed to obtain a diverse study population, and there was a wide age range at the time of the interview and years since transplantation, which is key when exploring a topic. Second, the research team consisted of people with different professional backgrounds, and their different perspectives helped in identifying and interpreting the various themes and subthemes. Third, the interviewer was independent from the care team of the participants. This made it easier for the participants to feel free in openly sharing and discussing their feelings and experiences without fear of consequences for their care. Some limitations of this study need to be considered as well. First, some of the patients indicated that they did not know whether particular psychosocial experiences were fully attributable to the HSCT, or (also) to other conditions or life events. Second, the COVID‐19 pandemic compelled us to hold the interviews digitally, which may have had some influence on the flow of the interview. Third, over the years, treatment has been optimized. Patients undergoing transplants now may experience different psychosocial effects than the patients included in our study, of whom a substantial number had undergone transplantation in an earlier time period. Finally, due to the long time since undergoing HSCT, there is a possibility of recall bias.

## 5 CONCLUSION

This study reveals four main life areas in which patients experience the psychosocial impact of the HSCT. A number of clinical practice recommendations for improving healthcare can be formulated based on this study. Patients may not have been offered supportive care services after discharge. We recommend the initiation of individualized medical support directly after discharge.^25^, ^27^ Furthermore, making medical support an addressed topic at the hospital check‐ups will add value in post‐HSCT supportive care initiation, for example, using clear validated patient‐reported outcome measures to initiate conversation about medical support. In future research, the long‐term psychosocial impact of paediatric HSCT for nonmalignant diseases should be studied among larger patient samples and their caregivers, ideally in a multicentre setting. This would allow identification of priorities for psychosocial support. The present study clearly points to a need to integrate pre‐emptive psychosocial support in the multidisciplinary care pathways during HSCT treatment and follow‐up for children with nonmalignant diseases.

## AUTHOR CONTRIBUTIONS

Joëll E. Bense approached the participants and collected hematopoietic stem cell transplant data. Lieke ter Welle conducted the interviews. Joëll E. Bense, Lieke ter Welleand  Arwen H. Pieterse, Anne P. J. de Pagter prepared the topic guide and analysed the data. Marieke Schimmel provided expert opinion. Joëll E. Bense and Lieke ter Welle wrote the manuscript. All authors critically revised the manuscript and approved the final version to be published.

## CONFLICT OF INTEREST

The authors declare no conflict of interest.

## Supporting information

Supplementary information.

## Data Availability

The data that support the findings (thematic coding matrices) of this study are available from the corresponding author, (Anne P.J. de Pagter), upon reasonable request. Due to the nature of this study, participants of this study did not agree for their data (e.g., full transcripts) to be shared publicly, so these data are not available.
