# Supplementary material for: Exploring the long‐term psychosocial impact of paediatric haematopoietic stem cell transplantation for nonmalignant diseases
Source: Health Expect. 2022 Jul 29;25(5):2462–70. doi: 10.1111/hex.13565 (PMC9615066; doi:10.1111/hex.13565)
Supplement: Supplementary file 1 — Supplementary information. [file HEX-25--s001.docx]

**Supplementary Information**

Supplemental Table S1. Topic guide

| **Briefly evaluate disease history of the patient** | |
| --- | --- |
|  | In [year of HSCT] you received a stem cell transplantation for [name disease] |
|  | Can you tell me something about that? |
| **Currently: experience and impact of the HSCT** | |
|  | How are you now? |
|  | What role does the transplantation play in your life? |
| **Future** | |
|  | How do you see the future? |
|  | Does the transplantation play a role in your future? |
|  | If yes: Can you tell something about that? |
| **Past (optional)** | |
|  | When you look back on the transplantation, what comes to mind? |
|  | How did your recovery go? |
|  | Decision for transplantation: How did you experience the decision for transplantation? |
| **Is there anything else about the time after your transplantation that we haven’t covered yet?** | |
| **Thank the patient for their time and trust** | |

Supplemental Table S2. Ten steps of Qualitative Analyses Guide of Leuven^1^

| **Stage** | **Process** |
| --- | --- |
| **1** | Thorough (re)reading of the interviews |
| **2** | Narrative interview report |
| **3** | From narrative report to conceptual interview scheme |
| **4** | Fitting-test of the conceptual interview schemes |
| **5** | Constant comparison process |
| **6** | Draw up a list of concepts |
| **7** | Coding process – back to the ‘ground’ |
| **8** | Analysis and description of concepts |
| **9** | Extraction of the essential structure |
| **10** | Description of the results |

Supplemental Table S3. Consolidated criteria for reporting qualitative studies (COREQ): 32-item checklist^2^

| ***No - Item*** | ***Guide question*** | ***Description*** |
| --- | --- | --- |
| **Domain 1: Research team and reflexivity** | | |
| Personal Characteristics | | |
| 1. Interviewer/facilitator | Which authors conducted the interview or focus group? | Page 4, study design: second author (LtW). |
| 2. Credentials | What were the researcher’s credentials? | 1^st^ author: MSc  2^nd^ author: BSc  3^rd^ author: RN - MSc  4^th^ author: MSc  5^th^ author: MSc  6^th^ author: MD – PhD  7^th^ author: MD – PhD  8^th^ author: MD - PhD |
| 3. Occupation | What was their occupation at the time of the study? | 1^st^ author: PhD student  2^nd^ author: Master student  3^rd^ author: Pediatric hematology nurse and PhD-student  4^th^ author: Healthcare psychologist  5^th^ author: General internal medicine specialist  6^th^ author: Pediatrician immunologist / Professor  7^th^ author: Associate professor doctor-patient decisions  8^th^ author: Pediatrician / hematologist |
| 4. Gender | Was the researcher male or female? | Page 4, study design: female. |
| 5. Experience and training | What experience or training did the researcher have? | Page 4, study design: BSc. |
| Relationship with participants | | |
| 6. Relationship established | Was a relationship established prior to study commencement? | Page 4, study design: no. |
| 7. Participant knowledge of the interviewer | What did the participants know about the researcher? | Page 4, study design: independent researcher. |
| 8. Interviewer characteristics | What characteristics were reported about the interviewer/facilitator? | Page 4, study design: independent researcher, who did not have any (treatment) relationship with the participants. |
| **Domain 2: study design** | | |
| Theoretical framework | | |
| 9. Methodological orientation and Theory | What methodological orientation was stated to underpin the study? | Page 4, study design: qualitative interview study.  Page 5, data analysis: thematic analysis based on the Grounded Theory. |
| Participant selection | | |
| 10. Sampling | How were participants selected? | Page 5, population and recruitment: purposive sample. |
| 11. Method of approach | How were participants approached? | Page 5, sample: participants were approached by telephone [JB] and had received complete study information. |
| 12. Sample size | How many participants were in the study? | Page 6, results: eighteen participants. |
| 13. Non-participation | How many people refused to participate or dropped out? Reasons? | Page 6, results: four participants refused to participate and did not provide any reason. |
| Setting | | |
| 14. Setting of data collection | Where was the data collected? | Page 4, data collection: videoconference. |
| 15. Presence of non-participants | Was anyone else present besides the participants and researchers? | Page 6, results: one participant preferred company from an adult caregiver. |
| 16. Description of sample | What are the important characteristics of the sample? | Page 6, results: 14 participants, HSCT during childhood, age range 14-49 years, median age 12 year after HSCT, equal gender distribution. Table 1, listed in characteristics table. |
| Data collection | | |
| 17. Interview guide | Were questions, prompts, guides provided by the authors? Was it pilot tested? | Table S1, interview topics.  Page 4, study design: semi-structured interviews with open ended questions. The topic list was evaluated and adjusted during the process. |
| 18. Repeat interviews | Were repeat interviews carried out? If yes, how many? | None |
| 19. Audio/visual recording | Did the research use audio or visual recording to collect the data? | Page 5, study design: interviews were video recorded. |
| 20. Field notes | Were field notes made during and/or after the interview or focus group? | Page 5, study design: field notes were taken about the researchers' reflections on the interview themes |
| 21. Duration | What was the duration of the interviews or focus group? | Page 6, results: median interview duration was 35 minutes (range 27-57) |
| 22. Data saturation | Was data saturation discussed? | Page 4, study design: data collection continued until data saturation was reached, which was defined as no new findings emerging in the analysis of the three latest consecutive interviews. Data saturation was reached after 14 interviews. |
| 23. Transcripts returned | Were transcripts returned to participants for comment and/or correction? | No. |
| **Domain 3: analysis and findings** | | |
| Data analysis | | |
| 24. Number of data coders | How many data coders coded the data? | Page 5, data analysis: four (LtW, JB, AhP, AdP) |
| 25. Description of the coding tree | Did authors provide a description of the coding tree? | No. |
| 26. Derivation of themes | Were themes identified in advance or derived from the data? | Page 6, results: derived from the data. |
| 27. Software | What software, if applicable, was used to manage the data? | Page 6, data analysis: ATLAS.ti. |
| 28. Participant checking | Did participants provide feedback on the findings? | No. |
| Reporting | | |
| 29. Quotations presented | Were participant quotations presented to illustrate the themes / findings? Was each quotation identified? | Page 6-12, results: Table 2-5, illustrative quotes. |
| 30. Data and findings consistent | Was there consistency between the data presented and the findings? | Page 6-12, results: Table 2-5, illustrative quotes. |
| 31. Clarity of major themes | Were major themes clearly presented in the findings? | Page 6, results: 1) Doing Okay, 2) Experiencing persisted involvement with healthcare services, 3) Influence on relationships with loved ones, and 4) Impact on participant’s life course. |
| 32. Clarity of minor themes | Is there a description of diverse cases or discussion of minor themes? | Page 6-12, results: Table 2-5, illustrative quotes. |

**References**

1. Dierckx de Casterlé B, Gastmans C, Bryon E, Denier Y. QUAGOL: a guide for qualitative data analysis. *Int J Nurs Stud*. Mar 2012;49(3):360-71. doi:10.1016/j.ijnurstu.2011.09.012

2. Tong A, Sainsbury P, Craig J. Consolidated criteria for reporting qualitative research (COREQ): a 32-item checklist for interviews and focus groups. *Int J Qual Health Care*. Dec 2007;19(6):349-57. doi:10.1093/intqhc/mzm042
